# Impact of “Killer Immunoglobulin-Like Receptor /Ligand” Genotypes on Outcome following Surgery among Patients with Colorectal Cancer: Activating KIRs Are Associated with Long-Term Disease Free Survival

**DOI:** 10.1371/journal.pone.0132526

**Published:** 2015-07-16

**Authors:** Kemal Beksac, Meral Beksac, Klara Dalva, Ergun Karaagaoglu, M. Bulent Tirnaksiz

**Affiliations:** 1 Department of General Surgery, Hacettepe University, Ankara, Turkey; 2 Department of Hematology, Ankara University, Ankara, Turkey; 3 Department of Biostatistics, Hacettepe University, Ankara, Turkey; Centro di Riferimento Oncologico, IRCCS National Cancer Institute, ITALY

## Abstract

Approximately 30 % of patients with stage II/III colorectal cancer develop recurrence following surgery. How individual regulation of host mediated anti-tumor cytotoxicity is modified by the killer-cell immunoglobulin-like receptor (KIRs) genotype is essential for prediction of outcome. We analyzed the frequency of KIR and KIR ligand Human Leukocyte Antigen Class I genotypes, and their effects on recurrence and disease-free survival (DFS). Out of randomly selected 87 colorectal cancer patients who underwent R0 resection operations between 2005 and 2008, 29 patients whose cancers progressed within a median five-year follow-up period were compared with 58 patients with no recurrence within the same time period. Recurrent cases shared similar tumor stages with non-recurrent cases, but had different localizations. We used DNA isolated from pathological archival lymphoid and tumor tissues for KIR and KIR ligand (HLA-C, group C1, group C2, and HLA-A-Bw4) genotyping. Among cases with recurrence, KIR2DL1 (inhibitory KIR) and A-Bw4 (ligand for inhibitory KIR3DL1) were observed more frequently (p=0.017 and p=0.024); and KIR2DS2 and KIR2DS3 (both activating KIRs) were observed less frequently (p=0.005 and p=0.043). Similarly, in the non-recurrent group, inhibitory KIR-ligand combinations 2DL1-C2 and 2DL3-C1 were less frequent, while the activating combination 2DS2-C1 was more frequent. The lack of KIR2DL1, 2DL1-C2, and 2DL3-C1 improved disease-free survival (DFS) (100% vs. 62.3%, p=0.05; 93.8% vs. 60.0%, p=0.035; 73.6% vs. 55.9%, p=0.07). The presence of KIR2DS2, 2DS3, and 2DS2-C1 improved DFS (77.8% vs. 48.5%, p=0.01; 79.4% vs. 58.5%, p=0.003; 76.9% vs. 51.4%, p=0.023). KIR2DS3 reduced the risk of recurrence (HR=0.263, 95% CI = 0.080-0.863, p=0.028). The number of activating KIRs are correlated strongly with DFS, none/ one/ two KIR : 54/77/98 months (p=0.004). In conclusion the inheritance of increasing numbers of activating KIRs and lack of inhibitory KIRs, independent of tumor localization or stage, is associated with long-term DFS.

## Introduction

Colorectal cancer is the third most common cancer in the world today. It can be cured with early diagnosis and successful surgical intervention [1–4]. The prognosis is largely dependent on the resectability and stage of the disease. Local recurrence has a powerful effect on survival and is observed in approximately %30 of cases. Recently, mutation analysis and gene expression profiling have been used to define more aggressive tumor types [2].

Host-mediated factors such as immune mechanisms, including natural killer (NK) cells, are also gaining importance in colorectal cancer research. NK cells exert their activity through killer-cell immunoglobulin-like receptors (KIRs) and their cognate ligands, including human leukocyte antigen [5] class I molecules. KIRs can play either activating or inhibiting roles [6,7]. The subcommittee of World Health Organization Nomenclature Committee has defined the KIR genes according to their function and structure [8]. The KIR genes have either two or three extracellular immunoglobulin domains, called 2D or 3D and either a long (L) or a short (S) intracellular domain. A total of fourteen different KIRs have been identified. Seven KIRs, KIR2DL1, KIR2DL2, KIR2DL3, KIR2DL5, KIR3DL1, KIR3DL2 and KIR3DL3 generate an inhibitory signal upon ligand binding. An activating signal is generated by six KIRs, KIR2DS1, KIR2DS2, KIR2DS3, KIR2DS4, KIR2DS5 and KIR3DS1. KIR2DL4 is able to generate both inhibitory as well as activating signals upon binding of its ligand [9]. The alleles of the HLA-C locus can be categorized into two groups of KIR ligands—group C1 and group C2—that can bind both activating and inhibiting KIRs [10]. HLA-C group 1 with asparagine at position 80 provides the ligand for KIR2DL2 and KIR2DL3, whereas HLA-C group 2 with lysine at position 80 provides the ligand for KIR2DL1.

The role of NK cells in the progression of precancerous lesions to colorectal cancer and tumor microenvironments have been reported in various studies [11–17]. Activating KIRs are generally claimed to be responsible for protection, while inhibitory KIRs are responsible for progression. There are only a few case-control studies that evaluate the role of KIRs on carcinogenesis [18,19], and these are inconclusive. Recently, a study on metastatic colorectal cancer evaluated the role of KIRs in responses to chemotherapy and survival [20]. We designed our research as a longitudinal study that compared the KIR genotypes of individuals diagnosed with colorectal cancer who exhibited long-term, disease-free survival, with the KIR genotypes of individuals who suffered local or distant recurrence of cancer. Our aim was to investigate the KIR / KIR ligand repertoire of each individual and if the genotype has any influence on cancer recurrence.

## Materials and Methods

### Patients, controls and treatment

In our study, we screened patients who were diagnosed with colorectal cancer and who underwent resection surgery between 2005 and 2008 at Hacettepe University, Department of General Surgery, in Ankara, Turkey. All patients who had standard predictors of worse outcome, such as R1-2 resections, palliative surgery, tumors presenting with adjacent organ invasion, post-operative anastomotic leakage were excluded. We also excluded patients who were younger than 18 years old, and who had synchronous metastasis, or who were immunosuppressed, lost to follow-up, or receivers of neo-adjuvant therapy. All remaining patients had R0 resection and had been given the appropriate adjuvant therapy [21]. Only cases with histo-pathologic diagnosis of adeno cancer were included in the study. 105 cases were found eligible initially, based on clinical parameters, but 18 of these were excluded from the study, because sufficient tumor/lymphoid tissue could not be retrieved from paraffin blocks. 87 patients qualified for the study. Among these, 29 patients manifested local or distant metastatic cancer recurrence during the study period of five years and formed Group 1. 58 patients who remained disease-free (non-recurrent cases) during the same five-year follow-up period formed Group 2. We defined disease-free survival (DFS) as the length of time between the date of initial therapy and the earliest date of tumor progression, death due to other reasons, or last follow-up.

As a control group KIR and KIR ligand data of 154 healthy potential related/unrelated hematopoietic stem cell donors aged 18–59 and screened for HLA and KIR genotyping were included into the study[22]. All controls had KIR and HLA-ABDRB1 typing available. 111 had HLA-C data available for Group C1 and C2 assessments. A-Bw4 assessment was possible among 136 of these control subjects.

This study was approved by the Institutional Ethical Committee of Hacettepe University (GO 13/94).

### DNA extraction, KIR and KIR ligand genotyping

We extracted DNA from lymph nodes and tumor material by using the QIAamp DNA FFPE Tissue Kit (QIAGEN, Hilden, Germany). The amount of DNA was adjusted to a concentration of 80ng/μL for analysis. We performed KIR genotyping on the DNA extracted from lymph nodes. For KIR ligand genotyping, we used the DNA extracted from the excised tumor paraffin sections. The Olerup SSP KIR Genotyping Kit (Olerup, Stockholm, Sweden) enabled us to detect 16 KIRs: KIR2DL1, 2DL2, 2DL3, 2DL4, 2DL5A/B, 2DS1, 2DS2, 2DS3, 2DS4, 2DS5, 3DL1, 3DL2, 3DL3, 3DS1, 2DP1, 3DP1. The Olerup KIR HLA Ligand Detection Kit was used to type HLA-A^Bw4+^, HLA-B^Bw4+Thr80^, HLA-B^Bw4+Ile80^, HLA-B^Bw4+Asp77,Thr80^, HLA-C^Asn80^, and HLA-C^Lys80^. Upon completion of amplification, we performed agarose gel electrophoresis to visualize the amplicons and to detect positive reactions.

### Statistical Analysis

The Statistical Package for the Social Sciences (IBM SPSS Statistics, USA) was used for data analysis. Pearson’s chi-square test and Fisher’s exact test were applied for categorical variables. For continuous variables, t-test was applied. Variables found positive in all subjects of both groups were excluded from t-test and univariate analysis. The possible factors identified in univariate analysis were further entered into Cox regression analysis, with backward selection, to determine independent predictors of survival. Among correlated factors with similar effects on survival, only those with clinical significance were selected. The proportional hazards assumption and model fit were assessed by means of residual (Schoenfeld and Martingale) analysis. Kaplan-Meier survival analysis and log rank tests were used for comparison of DFS.

## Results

### Patient Characteristics

Group 2 (non-recurrent cases) included more patients with right-sided colon and rectum tumors, but the difference was not significant (Table 1). In Group 1, recurrence was detected after 32.5 ± 30.5 (4.9–106.6) months of observation. Group 2 (the non-recurrent group) was observed for a mean of 60.2 ± 12.5 (24.2–84.7) months. Recurrence was observed in 33.3% of patients: on the site of anastomosis in 16 cases (18.4%) and with distant metastasis in 13 cases (14.9%).

**Table 1:** Characteristics of patients.

	Recurrent patients (n = 29)	Non-recurrent patients (n = 58)	P value
Age	59.5 ± 13.3	58.6 ± 12.3	n.s.
Gender (male/female)	14/15	32/26	n.s.
Adjuvant Therapy	23	37	n.s.
Tumor stage I/II/III	5/6/18	11/24/23	n.s.
Tumor Localization			
Right Colon	3	10	n.s.
Left Colon	6	7	
Sigmoid Colon	11	16	
Rectum	9	25	

n.s.: non significant

### KIR and KIR ligands

Analysis of individual KIR genotypes between groups showed that KIR2DL4, 3DL2, and 3DL3 were present in every patient. KIR3DL1 was present in more than 96% of patients. As seen in Table 2, KIR2DL1 was observed more frequently within the recurrent group regardless of local or distant metastasis (Table 3). KIRs 2DL2, 2DS2 and 2DS3 were observed more frequently among the non-recurrent patients. Analysis of individual KIR ligands within each group showed A-Bw4, the ligand for the inhibitory KIR3DL1, to be more frequent within the recurrent group. KIR ligand group C2 and group C1 frequencies were similar in both groups (Table 2). Group C1 (1/29 vs. 6/58, p = 0.09) or C2 (2/29 vs. 5/58, p = 0.36) homozygote frequencies did not differ between patient groups. Similarly, C1/C2 heterozygosity was observed among 73 (83.9%) of all patients and was equal between groups (26/29 vs. 47/58, p = 0.53).

**Table 2:** Comparison of KIR and KIR ligand genotype frequencies between patients recurring vs. those who did not.

KIR	Recurrent cases (n = 29)	Non-recurrent cases (n = 58)	P Value
2DL1	29 (100%)	48 (82.7%)	0.017
2DL2	18 (62%)	50 (86.2%)	0.01
2DL3	16 (55.2%)	23 (39.7%)	0.17
2DL5A/B	18 (62%)	35 (60.3%)	0.877
2DS1	14 (48.3%)	26 (44.8%)	0.761
2DS2	12 (41.4%)	42 (72.4%)	0.005
2DS3	7 (24.1%)	27 (46.5%)	0.043
3DS1	14 (48.3%)	21 (36.2%)	0.279
Group C1	27 (93.1%)	53 (91.4%)	0.571
Group C2	28 (96.6%)	52 (89.7%)	0.252
A-Bw4	24 (82.6%)	34 (58.6%)	0.024
2DL1-group C2	28 (96.6%)	43 (74.1%)	0,011
2DL2-group C1	17 (58.6%)	47 (81%)	0,025
2DL3-group C1	15 (51.7%)	19 (32.8%)	0,087
2DS1-group C2	13 (44.8%)	24 (41.4%)	0,759
2DS2-group C1	12 (41.4%)	40 (69%)	0,013
3DS1-Bw4	13 (44.8%)	19 (32.8%)	0,193
C2/ inhib KIR (+)*	15 (51.7%)	23 (39.7%)	0.087
C1/ inhib KIR (+)*	15 (51.7%)	13 (22.4%)	0.021

*: Combination of Group C and any cognate inhibitory KIR

**Table 3:** Comparison of KIR/Ligand frequencies: local recurrence versus distant metastasis. The variables found significant after comparison of frequencies between recurrent vs. non-recurrent cases were used in this table.

	Local recurrence (n = 16)	Distant metastasis(n = 13)	P value
2DL1	16 (100%)	13 (100%)	1,0
2DL2	9 (56.3%)	9 (69.2%)	0,474
2DS2	4 (25%)	8 (61.5%)	0,047
2DS3	5 (31.3%)	2 (15.4%)	0,292
A-Bw4	14 (87.5%)	10 (76.9%)	0,396
2DS2-group C1	4 (25%)	8 (61.5%)	0,047

To evaluate the impact of KIRs and their cognate ligands, we performed the following comparisons: When both the inhibitory KIR2DL1 and its ligand HLA C2 were present, the rate of recurrence increased (Table 2). On the other hand, the KIR2DS2 and group C1 combination was associated with a decrease in recurrence. We couldn’t perform KIR-ligand analysis for KIR2DS3, because its HLA ligand hasn’t been defined yet. We also analyzed co-segregation of at least one inhibitory KIR and its ligand. We detected inhibitory KIRs regardless of Group C1 or C2 status more frequently within the recurrent group (Table 2).

The frequency of inhibitory KIR2DL2 in the non-recurrent group was an unexpected finding, so we performed further analysis, which revealed 2DS2 to co-segregate with KIR2DL2 invariably (data not shown in tables).

Although KIR2DS2 wasn’t observed very frequently (41.4%) within the recurrent group (Table 2), the presence of this activating KIR was associated with distant rather than local recurrence (61.5% vs. 25%, p = 0.047) (Table 3). All anonymous individual KIR and KIR ligand data can be accessed in the S1 and S2 Tables.

### Survival analysis

**Fig 1:**
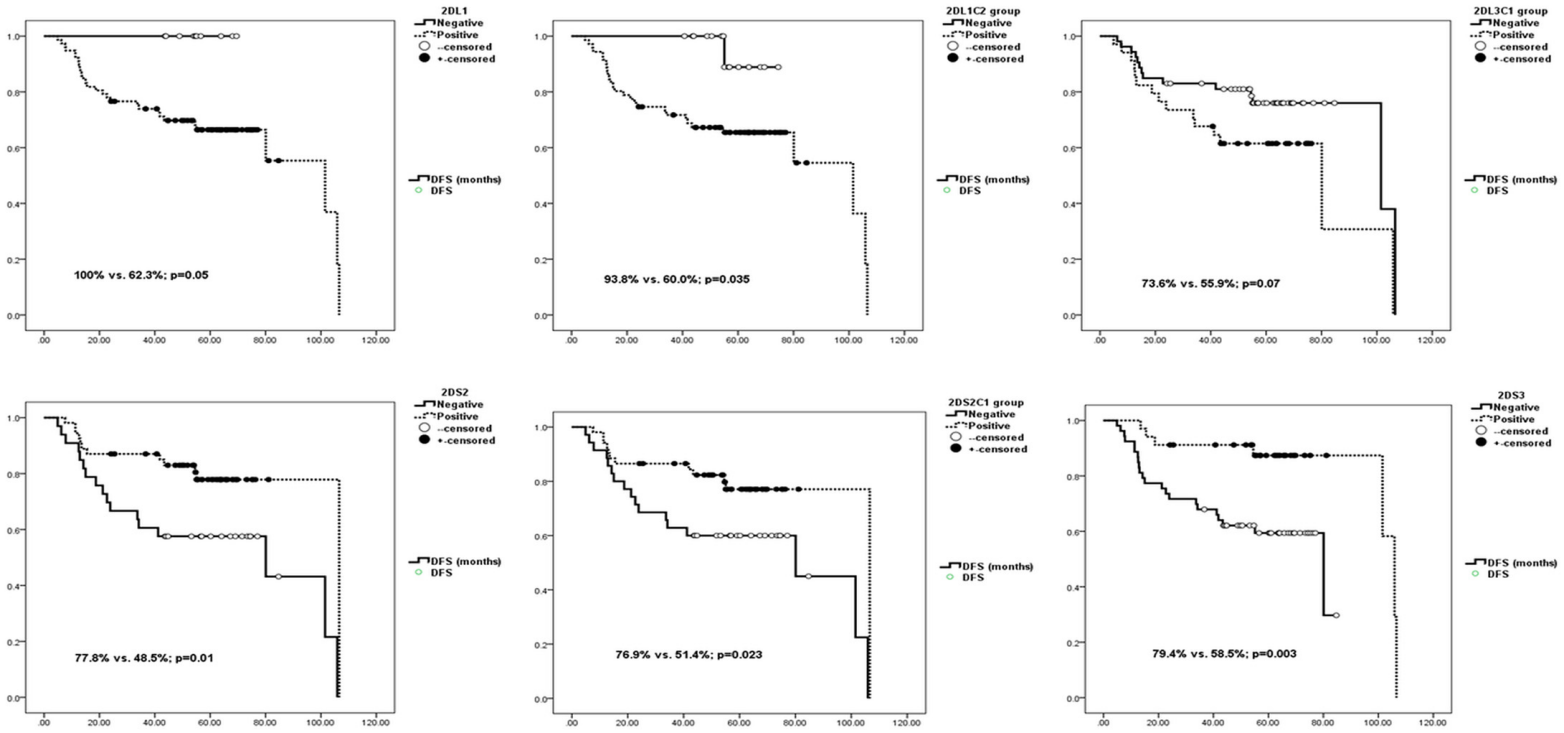
Impact of inhibitory (2DL1, 2DL1-Group C2 and 2DL3 Group C1) (upper row) and activating (2DS2, 2DS2- Group C1 and 2DS3) (lower row) KIRs on Progression Free Survival. X axis shows percentage of survivors without recurrence against months of follow-up (y axis). The lack of inhibitory KIR2DL1, 2DL1-C2, or 2DL3-C1 improved DFS (100% vs. 62.3%, p = 0.05; 93.8% vs. 60.0%, p = 0.035; 73.6% vs. 55.9%, p = 0.07). Presence of activating KIR2DS2, 2DS2-C1 and 2DS3 (77.8% vs. 48.5%, p = 0.01; 76.9% vs. 51.4%, p = 0.023; 79.4% vs. 58.5%, p = 0.003;) are also associated with longer DFS.

**Fig 2:**
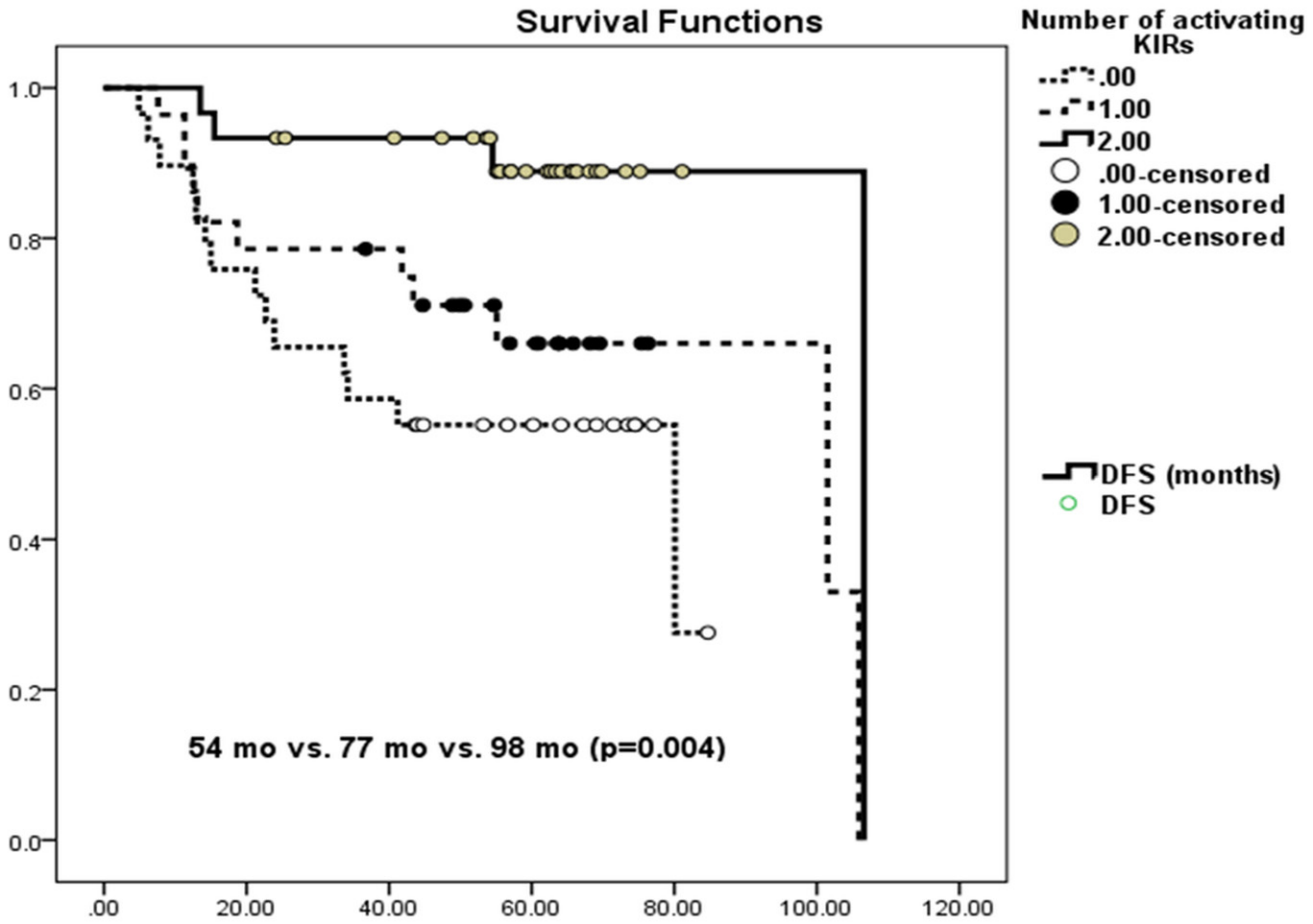
Influence of number of activating KIRs on Disease Free Survival: none vs. one vs. two activating KIRs. Patients who have two activating KIRs in their genotype have longer DFS compared to those who have one activating KIR. Patients who lack any activating KIR have the shortest DFS: no KIR: 54 months (CI: 42–65); one KIR: 77 months (CI: 61–92); two KIRs: 98 months (CI: 87–108) (p = 0.004). X axis shows percentage of survivors without recurrence against months of follow-up (y axis).

Kaplan-Meier DFS analysis confirmed the results obtained by frequency comparisons. The effect of activating KIRs appeared to be more significant than the effect of inhibitory KIRs: The lack of KIR2DL1, 2DL1-C2, or 2DL3-C1 improved DFS (100% vs. 62.3%, p = 0.05; 93.8% vs. 60.0%, p = 0.035; 73.6% vs. 55.9%, p = 0.07) (Fig 1), as did the presence of KIR2DS2, 2DS3, and 2DS2-C1 (77.8% vs. 48.5%, p = 0.01; 79.4% vs. 58.5%, p = 0.003; 76.9% vs. 51.4%, p = 0.023) (Fig 1). Hazard risk assessments of all the significant factors on DFS revealed that only 2DS3 reduced the risk of recurrence significantly (HR = 0.263, 95% CI = 0.080–0.863, p value = 0.028). The number of activating KIRs had the following effect on DFS: no KIR: 54 months (CI: 42–65); one KIR: 77 months (CI: 61–92); two KIRs: 98 months (CI: 87–108) (p = 0.004) (Fig 2). Likewise after adding more activating KIR (none vs. one vs. two vs. three KIRs) comparison was still significant (p = 0.004).

### KIRs and clinical parameters

We also analyzed double-activating KIR (2DS2 and 2DS3) positivity according to tumor localization. In the non-recurrent group, the following percentage of cancer cases had this double-activating KIR expression: 10/25 (40%) for rectum cancer, 5/16 (31.3%) for sigmoid cancer, 7/10 (70%) for right-sided colon cancer, and 4/7 (57.1%) for left-sided colon cancer. We observed similar ratios among the recurrent group: 1/9 (11.1%) for rectum cancer and 3/11 (27.3%) for sigmoid cancer.

Among stage I cases, the activating KIR expression was observed more frequently among non-recurrent vs. recurrent patients (7/11 vs. 2/5) without statistical significance. The double-activating KIR expression was more frequent in the non-recurrent group when clinical stages were grouped accordingly. Frequency of double activating KIR (non-recurrent vs. recurrent patients) positive cases within in each stage is as follows: stage I: 4/11 vs. 0/5; stage II: 10/24 vs. 0/6; stage III: 12/23 vs. 4/18.

Twelve out of thirteen right-sided colon tumors were stage II or III, and double-activating KIR expression was not detected in any of the recurrent cases.

### KIRs and Normal Subjects

In an attempt to analyze the frequency of these significant inhibitory and activating KIRs among normal subjects we compiled KIR data from a pool of healthy donors analyzed for KIR genotypes to match for KIR ligands prior to hematopoietic stem cell transplantation. The comparison of the controls vs. patients with colorectal cancer is summarized in Table 4. Activating KIR frequencies (either alone or combined) were similar between controls and patients. However inhibitory KIRs (2DL2) alone or with the cognate ligand (2DL2-Group C1; 2DL1-Group C2) or the KIR ligand alone (A-Bw4) were more frequent among patients.

**Table 4:** Comparison of KIRs and KIR ligands between controls and patients with Colorectal Cancer. Only the activating and inhibitory KIRs and ligands found to be associated with recurrence are included into the analysis.

	Control	Colorectal Cancer	
	(n:154)	(n:87)	P
2DS1	75/154 (48.7%)	40/87 (45.97%)	0,68
2DS2	103/154 (66.88%)	54/87 (62.07%)	0,45
2DS3	52/154 (33.76%)	34/87 (39.08%)	0,41
(2DS1+2DS2+2DS3)	23/154 (14.93%)	16/87 (18.39%)	0,26
(2DS1+2DS2+3DS1)	43/154 (27.92%)	20/87 (22.98%)	0,40
(2DS1+2DS2+2DS3+3DS1)	20/154 (12.98%)	12/87 (13.79%)	0.86
(2DS1+2DS2)	54/154 (35.06%)	28/87 (32.18%)	0,65
(2DS1+2DS3)	25/154 (16.23%)	16/87 (18.39%)	0,67
(2DS2+2DS3)	49/154 (31.82%)	30/87 (34.48%)	0,67
3DS1	76/154 (49.35%)	35/87 (40.22%)	0,17
2DL1	147/154 (95.45%)	77/87 (88.5%)	**0,04**
2DL2	103/154 (66.88%)	68/87 (78.16%)	**0,001**
2DL2-groupC1	64/111 (57.65%)	64/87 (73.56%)	**0,02**
2DL1-groupC2	78/111 (70.27%)	71/87 (81.61%)	0,067
2DS2-GroupC1	63/111 (56.76%)	52/87 (59.77%)	0,67
A-Bw4	63/136 (46.32%)	58/87 (66.6%)	**0,003**

## Discussion

Colon cancer pathological staging has failed to predict recurrence precisely [2]. Malignancy-associated features (i.e., aggressive vs. indolent cancer) can determine clinical outcome. Genetic expression profiling is a promising tool for predicting disease-free survival rates for colorectal cancer patients [2]. In our study, clinical variables such as age, gender, clinical stage, and adjuvant treatment were similar across the two (recurrent and non-recurrent) patient groups.

The loss of HLA Class I expression in colon cancer cells is one of the defense mechanisms leading to escape from cytotoxic lymphocyte mediated effects and increasing the role of NK cells [23]. In our study, we successfully showed that KIR ligand genotypic expressions, alone or together with KIR, play a role in disease recurrence. Our approach enabled robust differential genotyping of HLA Class I compared to immuno-histochemical staining, which is less descriptive. Although genotypic analysis does not guarantee cellular expression of these ligands, the simultaneous presence of both group C1 and C2 ligands in our study supports the absence of a haplotype or bi-allelic loss of HLA.

As evidence in favor of our hypothesis, two of the activating KIRs—2DS2 and 2DS3—were observed more frequently in patients whose disease did not progress during the observation period. This study, for the first time, found that the number of activating KIRs is associated with longer DFS: 54 months (no activating KIRs) vs. 98 months (two activating KIRs). Our study is not the first to suggest the protective role of increased number of activating KIR genes. In an earlier study, Verheyden et al reported the probability of relapse at 5 years to be significantly lower for patients with acute leukemia who received a graft from a donor with the 2DS1(+)2DS2(+) genotype than for those who received a transplant from other donors (17 vs. 63%, respectively; P = 0.018)[24]. Furthermore, we were able to show that the protective effect of 2DS2 and 2DS3 is independent of tumor stage or localization. Even within a group of 13 patients diagnosed with right-sided colon localization, double-activating KIR expression was associated with lack of recurrence.

In addition to observing the effects of activating KIRs, we found that one of the inhibitory KIRs (2DL1) was present in all patients with recurrent cancer, but appeared less frequently in non-relapse cases. As an unexpected result of our study, we observed an inhibitory KIR (2DL2) more frequently in non-recurrent patients. To further elucidate this observation, we evaluated KIRs that share the same group C1 as ligands, and found a strong association between the activating KIR2DS2 and the inhibitory KIR2DL2. Another inhibitory KIR that shares the same ligand as 2DL2 is 2DL3. KIR2DL3 is associated with recurrent disease only when patient has HLA group C1 and lacks KIR2DS2 and 2DL2.

By evaluating KIR ligands alone, we detected only one significant result: A-Bw4 was associated with recurrent disease. A-Bw4 is the ligand for the inhibitory KIR3DL1, which is invariably present in all subjects. To further evaluate the KIR and KIR ligand interactions, we analyzed individual KIR-KIR ligand combinations and found that KIR2DL1-group C2 (and to a lesser extent, 2DL3-group C1) combinations were associated with recurrence. Conversely, we observed KIR2DL2-group C1 and KIR2DS2-group C1 combinations more frequently in non-recurrent patients. A recent study reported that the KIR2DL1 ligand group C2 predisposes patients with pediatric B-ALL to relapse, and is correlated with the number of alleles. In the same study, group C1 homozygosity was found to be protective for this type of leukemia [25]. In our study, the majority of patients were group C1/C2 heterozygotes. This distribution prevented the progression-related effects of groups C1 and C2.

The potency of inhibition compared to the activating effects of NK-mediated immune modulation is still controversial. The current literature suggests that inhibitory effects are more dominant, and activating effects are effective only in the absence of inhibitory KIRs [26,27]. In our study, for the first time, we observed that the absence of inhibitory KIRs together with the presence of activating KIRs are associated with less recurrence: Among all inhibitory KIRs, only those that share the ligand group C1 are significantly associated with recurrence.

Among relapsing patients, the combination of activating KIR2DS2-C1 was observed less frequently than those who remained disease free. When we did observe this genotype in recurrent patients, it was associated with distant metastasis rather than recurrence at the anastomosis site. When the radio-resistance of NK cells and the increase of NK-mediated effects following radiotherapy are taken into account, protective effects within adjacent tissues is a possibility [28].

We found two other studies that analyzed KIR and KIR ligands in patients with colorectal cancer [18,20]. In the first study published by Middleton and colleagues they compared patients with healthy controls, but were not able to observe any differences in KIR/KIR ligand frequencies [18,19]. In our study we were able to find, for the first time, an increased frequency of inhibitory KIRs (2DL2) or ligands (A-Bw4) alone or in the presence of their cognate ligands (2DL2-Group C1 and 2DL1-Group C2) among patients with colorectal cancer compared to healthy donors. Conversely activating KIRs were similarly distributed. 2DL1 is a KIR, widely distributed among healthy subjects but less frequent frequency of the cognate ligand, may limit its effects.

The second study was authored by De Re et al. (16). They screened 224 patients diagnosed with stage IV recurrent colorectal cancer who were receiving chemotherapy, with or without resection surgery, for KIR and HLA ligands. They found that the presence of activating KIRs (2DS5, 2DS1, 3DS1, and KIR3DS1/HLA-Bw4-I80) and inhibitory KIRs (2DL5A) was associated with complete response (CR) to chemotherapy. Conversely, the absence of KIR2DS4 and 3DL1 was associated with CR. They observed an increasing number of activating KIR genes (with their cognate ligands) to be associated with better response. Although they were able to observe the effect of KIRs and KIR ligands on overall survival, they could not detect any association between KIR/ligands and time to progression. Although their study differs from ours with respect to patient selection and treatment, there are similarities between the two studies that support the anticancer effects of activating KIRs. In our study, activating KIRs are associated with long-term response. Our study is the first to analyze KIR/ ligands in stage I-IV patients who were treated by surgery followed by adjuvant therapy. This approach enabled us to compare patients who were possibly cured with patients whose cancers progressed despite surgical resection. Except in our study such a model has not been attempted in any of the solid tumors yet. Our study also provides evidence that the activating KIRs, KIR2DS2 (alone or with the presence of the ligand group C1) and KIR2DS3 are associated with protection from recurrence. The presence of KIR2DS3 reduced the risk of recurrence significantly (HR: 0.263). We were also able to associate the inhibitory KIR 2DL1 and ligand A-Bw4 with recurrence. Our findings support the occurrence of anti-tumor NK-mediated immune response within this population of surgically treated patients.

Another interesting finding is that although right-sided colon tumors have been associated with a worse prognosis than left-sided tumors [29], our non-recurrent group had almost identical frequency of right-sided and left-sided colon tumors (17.2% vs. 10.3%). The likely explanation for this is that 70% of right-sided colon tumors in the non-recurrent group had double-activating KIRs, which provided protection against recurrence.

In conclusion, our study, together with the two studies we cited (14, 16), provide evidence that activating KIRs play a protective role in cancer recurrence. However, KIR ligands belong to a whole range of molecules that were not included in this study. Furthermore, our hypothesis is based on genotyping results that are not necessarily associated with protein expression. Genotypic features associated with high proliferative or aggressive tumor behaviors may complicate the interactions. Nevertheless, activating KIRs 2DS2 and 2DS3 in the presence of their ligands suggest a genotype that protects against recurrence. We believe it would be valuable to expand this study to test patients who are more prone to recurrence.

## Supporting Information

S1 Table Table lists recurrent colorectal cancer patients’ individual KIR data. (PDF)

S2 Table Table lists non-recurrent colorectal cancer patients’ individual KIR data. (PDF)
