# Supplementary material for: Impact of “Killer Immunoglobulin-Like Receptor /Ligand” Genotypes on Outcome following Surgery among Patients with Colorectal Cancer: Activating KIRs Are Associated with Long-Term Disease Free Survival
Source: PLoS One. 2015 Jul 16;10(7):e0132526. doi: 10.1371/journal.pone.0132526 (PMC4504472; doi:10.1371/journal.pone.0132526)
Supplement: S1 Table — (PDF) [file pone.0132526.s001.pdf]

2D L1	2D L2	2D L3	2D L4	2DL5 A/B	3D L1	3D L2	3D L3	2D S1	2D S2	2D S3	2DS4 normal	2DS4 truncated	2D S5	3D S1	Tipleri	2D S1	C2 GRUP	C2 activation	2DL2	2D L3	2D S2	C1 GRUP	C1 activation	3D L1	3D S1	Bw4
-	+	+	+	+	+	-	+	+	-	+	-	-	B	B		0	1	0	1	0	1	1	2	1	0	10
-	+	+	+	+	+	-	-	+	-	+	-	-	B	B		0	1	0	1	0	0	1	0	1	0	9
+	+	+	+	+	+	-	-	+	+	+	+	+	B	B		0	1	0	0	1	0	1	0	1	1	7
+	+	+	+	+	+	+	+	-	+	-	+	+	B	B		1	1	2	1	1	1	1	2	1	1	7
+	+	-	+	+	+	-	-	-	+	-	-	-	A	A		0	1	0	0	1	0	1	0	1	0	10
+	+	-	+	+	+	-	-	-	-	+	-	-	A	A		0	1	0	0	1	0	1	0	1	0	7
+	+	-	+	+	+	-	-	-	+	+	-	-	A	A		0	1	0	0	1	0	0	3	1	0	9
-	+	-	+	+	+	-	-	-	-	+	+	-	B	B		0	1	0	1	0	0	1	0	1	0	4
+	+	+	+	+	+	+	-	-	-	+	+	+	B	B		1	1	2	0	1	0	1	0	1	1	10
-	+	+	+	+	+	-	+	+	+	+	-	-	B	B		0	1	0	1	0	1	1	2	1	0	10
-	+	+	+	+	+	+	-	-	-	+	+	+	B	B		1	1	2	1	0	0	1	0	1	1	4
+	+	-	+	+	+	-	-	+	-	-	-	-	B	B		0	1	0	0	1	0	1	0	1	0	3
+	+	+	+	+	+	+	-	-	-	+	+	+	B	B		1	1	2	0	1	0	1	0	1	1	4
+	+	-	+	+	+	-	-	-	+	+	-	-	A	A		0	1	0	1	1	0	1	0	1	0	4
+	+	+	+	+	+	+	+	-	-	+	+	+	B	B		1	1	2	1	1	1	1	2	1	1	3

-	+	-	+	+	+	-	+	-	-	+	-	-	**B**	**B**		0	1	0	1	0	1	1	2	1	0	10
+	+	+	+	+	+	+	+	-	-	+	+	+	**B**	**B**		1	1	2	1	1	1	1	2	1	1	4
-	+	+	+	+	+	+	+	+	-	+	-	+	**B**	**B**		1	1	2	1	0	1	1	2	1	1	10
+	+	+	+	+	+	+	-	-	-	+	+	+	**B**	**B**		1	1	2	0	1	0	1	0	1	1	0
-	+	+	-	+	+	+	+	-	-	-	+	+	**B**	**B**		1	1	2	1	0	1	1	2	0	1	4
-	+	-	+	+	+	-	+	-	-	+	-	-	**B**	**B**		0	1	0	1	0	1	1	2	1	0	3
-	+	+	+	+	+	+	+	+	-	+	+	+	**B**	**B**		1	1	2	1	0	1	1	2	1	1	10
+	+	-	+	+	+	-	-	-	-	+	-	-	**A**	**A**		0	1	0	0	1	0	1	0	1	0	7
-	+	-	+	+	+	-	-	-	-	+	-	-	**A**	**A**		0	1	0	1	0	0	1	0	1	0	7
+	+	-	+	+	+	-	-	-	-	+	-	-	**A**	**A**		0	1	0	0	1	0	1	0	1	0	4
-	+	+	+	+	+	+	-	-	-	-	+	+	**B**	**B**		1	1	2	1	0	0	0	3	1	1	13
+	+	+	+	+	+	+	+	-	-	+	+	-	**B**	**B**		1	1	2	1	1	1	1	2	1	0	3
-	+	+	+	+	+	+	+	-	-	-	+	+	**B**	**B**		1	0	3	1	0	1	1	2	1	1	4
+	+	+	+	+	+	+	-	-	+	-	+	+	**B**	**B**		1	1	2	0	1	0	1	0	1	1	4

universally present

2DS1 two different

**NOT :**	**0**	**absent**
	**1**	**present**

**NOT :**	**0***	**no activation expected**
	**1***	**Activation expected**

2	both activating and inhibitory
3	either KIR or ligand is missing

*	Ligand is HLA-G
**	Ligand is HLA-A*03, HLA-A*11
A-Bw4	HLA-A (A23, A24, A25, A32,...) Bw4
	is universally present

Supplemental Table-1: KIR and KIR ligand data of each individual who have had recurrence
