# Supplementary material for: Impact of “Killer Immunoglobulin-Like Receptor /Ligand” Genotypes on Outcome following Surgery among Patients with Colorectal Cancer: Activating KIRs Are Associated with Long-Term Disease Free Survival
Source: PLoS One. 2015 Jul 16;10(7):e0132526. doi: 10.1371/journal.pone.0132526 (PMC4504472; doi:10.1371/journal.pone.0132526)
Supplement: S2 Table — (PDF) [file pone.0132526.s002.pdf]

patient nun	age	gender	TNM	stage	localization
1	54	E	T3N0Mx	2	Rektum
2	74	E	T3N0Mx	2	Rektum
3	57	K	T3N1bMx	3	Sigmoid
4	66	E	T1N0Mx	1	Rektum
5	69	K	T3N0Mx	2	Rektum
6	51	K	T2N0Mx	1	Rektum
7	69	K	T3N0Mx	2	Sigmoid
8	47	K	T4aN2aMx	3	Rektum
9	77	K	T3N1bMx	3	Left Colon
10	65	E	T3N1aMx	3	Sigmoid
11	59	E	T1N0Mx	1	Left Colon
12	62	K	T1N0Mx	1	Left Colon
13	65	E	T3N2aMx	3	Left Colon
14	71	K	T3N0Mx	2	Right Colon
15	42	E	T1N0Mx	1	Sigmoid
16	53	K	T4aN1bMx	3	Left Colon
17	61	E	T3N0Mx	2	Right Colon
18	80	K	T3N0Mx	2	Sigmoid
19	77	K	T3N0Mx	2	Right Colon
20	60	E	T2N0Mx	1	Sigmoid
21	41	K	T4bN0Mx	3	Right Colon
22	54	E	T3N2bMx	3	Rektum
23	70	E	T2N0Mx	1	Right Colon
24	66	E	T3N1bMx	3	Sigmoid
25	44	E	T2N0Mx	1	Rektum
26	53	K	T2N0Mx	1	Sigmoid
27	36	K	T1N0Mx	1	Rektum
28	54	K	T1N0Mx	1	Rektum
29	56	E	T3N0Mx	2	Rektum
30	68	E	T3N0Mx	2	Rektum
31	47	E	T3N2bMx	3	Rektum
32	23	E	T3N2aMx	3	Rektum
33	51	K	T2N0Mx	2	Rektum
34	49	K	T3N0Mx	2	Rektum
35	43	E	T3N2bMx	3	Rektum

36	80	E	T3N0Mx	2	Rektum
37	70	E	T3N0Mx	2	Rektum
38	53	E	T4N0Mx	3	Rektum
39	33	K	T3N0Mx	2	Rektum
40	51	K	T3N1aMx	3	Sigmoid
41	47	K	T4N2bMx	3	Rektum
42	77	E	T2N0Mx	2	Rektum
43	58	K	T2N1aMx	3	Rektum
44	65	E	T3N0Mx	2	Left Colon
45	70	E	T3N0Mx	2	Right Colon
46	69	E	T3N0Mx	2	Right Colon
47	63	E	T3N1bMx	3	Sigmoid
48	47	K	T3N1bMx	3	Sigmoid
49	50	E	T3N0Mx	2	Left Colon
50	70	K	T3N0Mx	2	Right Colon
51	54	E	T3N0Mx	2	Right Colon
52	63	E	T3N1aMx	3	Right Colon
53	77	E	T3N0Mx	2	Right Colon
54	59	K	T2N1aMx	3	Sigmoid
55	72	K	T3N1bMx	3	Sigmoid
56	52	K	T3N0Mx	2	Sigmoid
57	52	E	T3N1bMx	3	Rektum
58	53	E	T4N1bMx	3	Sigmoid

operation dat	follow-up date	duration FU
12.03.2007	18.06.2012	64,2
24.04.2007	28.06.2013	75,2
14.05.2007	4.04.2011	47,4
12.07.2007	15.03.2013	69,1
17.09.2007	11.02.2013	65,8
7.01.2008	2.04.2013	63,7
20.09.2007	2.05.2011	44,0
27.09.2007	30.04.2013	68,1
8.01.2007	12.05.2010	40,7
8.02.2007	22.03.2013	74,5
24.05.2007	18.05.2009	24,2
6.07.2007	7.08.2009	25,4
3.08.2007	27.12.2012	65,8
10.08.2007	18.09.2012	62,2
31.08.2007	6.05.2011	44,8
21.09.2007	31.07.2012	59,2
24.09.2007	2.05.2013	68,2
7.11.2007	31.05.2012	55,6
19.06.2006	15.02.2013	81,1
7.08.2006	18.09.2012	74,5
11.09.2006	5.06.2012	69,8
27.11.2006	4.02.2013	75,4
7.12.2006	11.12.2012	73,2
3.02.2006	19.12.2011	71,5
28.03.2006	30.04.2010	49,8
9.05.2006	2.05.2011	60,6
11.05.2006	25.05.2012	73,5
22.05.2006	19.09.2012	77,1
25.05.2006	9.05.2013	84,7
21.07.2006	23.03.2012	69,1
13.10.2006	19.01.2012	64,1
13.11.2006	19.02.2013	76,3
7.01.2008	1.04.2013	63,7
25.01.2008	9.10.2013	69,5
18.02.2008	31.07.2013	66,3

25.02.2008	2.03.2011	36,7
4.04.2008	18.10.2012	55,3
12.05.2008	30.09.2013	65,6
6.06.2008	22.08.2013	63,4
20.06.2008	27.02.2013	57,1
21.07.2008	26.07.2013	61,0
4.08.2008	28.09.2012	50,5
17.11.2008	15.04.2013	53,7
17.01.2008	16.07.2012	54,7
7.02.2008	18.01.2013	60,2
16.02.2008	16.05.2013	63,9
24.03.2008	4.10.2013	67,3
7.08.2008	16.01.2013	54,1
8.08.2008	20.12.2012	53,2
1.09.2008	8.05.2013	57,0
18.09.2008	20.12.2012	51,8
22.10.2008	3.05.2013	55,1
6.12.2008	8.07.2012	43,7
24.01.2008	25.09.2011	44,7
20.03.2008	14.05.2013	62,7
17.07.2008	12.03.2013	56,6
7.09.2008	14.09.2012	48,9
23.10.2008	26.06.2013	56,9

	2DL1	2DL2	2DL3	2DL4	2DL5A/B
+	+	-	+	-	+
+	+	+	+	+	+
+	+	-	+	+	+
+	+	-	+	-	+
+	+	-	+	+	+
+	+	+	+	+	+
-	-	+	+	-	+
-	+	-	+	+	+
+	+	+	+	+	+
+	-	+	+	+	+
+	+	-	+	+	+
+	+	-	+	+	+
+	+	-	+	+	+
+	+	+	+	+	+
+	+	+	+	-	+
+	+	+	+	-	+
+	+	+	+	+	+
+	+	-	+	+	+
+	+	-	+	+	+
+	-	+	+	-	+
+	+	-	+	+	+
+	+	+	+	-	+
+	+	-	+	-	+
+	+	+	+	-	+
+	+	+	+	-	-
+	+	+	+	-	+
+	+	-	+	+	+
+	+	-	+	+	+
+	-	+	+	-	+
+	+	-	+	+	+
+	+	-	+	-	+
+	+	+	+	+	+
+	+	+	+	+	+
-	+	-	+	+	-
+	+	-	+	+	+

+	+	-	+	+	+
+	+	-	+	+	+
+	+	-	+	+	+
+	+	-	+	+	+
+	+	-	+	+	+
+	+	+	+	-	+
+	+	-	+	+	+
+	+	-	+	+	+
-	+	-	+	-	+
+	+	-	+	-	+
-	+	-	+	-	+
+	-	+	+	-	+
-	+	-	+	+	+
+	-	+	+	-	+
+	+	-	+	+	+
+	+	+	+	-	+
-	+	-	+	+	+
-	-	+	+	+	+
+	+	-	+	-	+
+	+	-	+	+	+
-	-	+	+	-	+
-	+	-	+	-	+
+	+	-	+	+	+

HER İNSANDA BULUNMASI GEREKENELER

2DS1'in iki farklı kuyuda farklı alt tipleri olduğunu gösterme

3DL1	3DL2	3DL3	2DS1	2DS2	2DS3	2DS4 normal	DS4 truncate	2DS5
+	+	+	+	+	-	+	-	-
+	+	-	+	+	-	+	-	-
+	+	+	+	+	+	+	+	-
+	+	-	-	-	-	+	-	-
+	+	-	+	+	-	+	-	-
+	+	+	+	-	-	+	+	-
+	+	-	-	-	+	+	-	-
+	+	-	+	+	-	+	-	-
+	+	-	+	+	+	+	+	+
+	+	+	-	-	+	-	+	+
+	+	+	+	+	+	-	+	+
+	+	-	+	+	-	+	-	-
+	+	+	+	-	-	-	+	+
+	+	+	+	+	-	+	-	-
+	+	-	-	-	+	+	-	-
+	+	+	+	+	-	+	+	-
+	+	+	+	-	-	+	+	+
+	+	+	+	+	-	+	+	+
+	+	+	+	+	-	-	+	+
+	+	-	-	-	-	+	-	-
+	+	-	+	+	-	+	-	-
+	+	-	+	-	-	+	-	-
+	+	+	+	+	+	+	+	+
+	+	+	-	-	-	+	-	-
+	+	-	+	-	-	+	-	-
+	+	-	+	-	-	+	-	+
+	+	+	-	-	+	-	+	-
+	+	+	-	-	-	+	+	+
+	+	-	-	-	-	+	-	-
+	+	-	+	+	-	+	-	-
+	+	-	-	-	-	+	+	-
+	+	-	+	-	-	+	-	+
+	+	+	+	-	-	+	+	-
+	+	+	+	-	-	+	+	-
+	+	-	+	+	+	+	-	-

+	+	+	+	-	-	+	+	+
+	+	-	+	+	-	+	-	-
+	+	-	+	+	-	+	-	-
+	+	+	+	+	-	+	+	+
+	+	+	+	+	-	-	+	+
+	+	-	+	-	+	+	-	-
+	+	-	+	-	+	+	-	-
+	+	-	+	+	-	+	-	+
+	+	-	+	-	-	+	-	-
+	+	+	-	-	-	+	+	+
+	+	-	+	-	-	+	-	-
+	+	-	-	-	+	-	-	-
+	+	+	+	+	-	-	+	+
+	+	-	-	-	+	+	-	-
+	+	+	+	+	-	+	+	+
+	+	-	+	+	-	-	-	-
+	+	+	+	+	-	-	-	+
+	+	+	-	-	-	-	+	+
+	+	-	+	-	-	+	-	-
+	+	+	+	+	-	+	-	+
+	+	-	-	-	+	+	-	-
+	+	-	+	-	+	+	-	-
+	+	-	-	+	+	+	-	-

k için

3DS1
B
B
B
A
B
B
A
B
B
B
B
B
B
B
A
B
B
B
B
A
B
B
B
B
B
B
B
B
A
B
A
B
B
B
B

2DL1	2DS1	C2 GRUP	C2 aktivasyon	2DL2	2DL3
1	1	1	2	1	0
1	0	1	0	1	1
1	1	1	2	1	0
1	0	1	0	1	0
1	0	1	0	1	0
1	1	1	2	1	1
0	0	1	3	0	1
0	0	1	3	1	0
1	0	0	3	1	1
1	1	0	3	0	1
1	1	1	2	1	0
1	0	1	0	1	0
1	1	1	2	1	0
1	1	1	2	1	1
1	0	1	0	1	1
1	1	1	2	1	1
1	1	1	2	1	1
1	1	1	2	1	0
1	1	1	2	1	0
1	0	1	0	0	1
1	0	1	0	1	0
1	0	1	0	1	1
1	1	1	2	1	0
1	1	1	2	1	1
1	0	1	0	1	1
1	0	1	0	1	1
1	1	1	2	1	0
1	1	1	2	1	0
1	0	1	0	0	1
1	0	1	0	1	0
1	0	1	0	1	0
1	0	1	0	1	1
1	1	1	2	1	1
0	1	1	1	1	0
1	0	1	0	1	0

B	1	1	1	2	1	0
B	1	0	1	0	1	0
B	1	0	1	0	1	0
B	1	1	1	2	1	0
B	1	1	1	2	1	0
B	1	0	1	0	1	1
B	1	0	0	3	1	0
B	1	0	1	0	1	0
B	0	0	1	3	1	0
B	1	1	0	3	1	0
B	0	0	0	3	1	0
A	1	0	1	0	0	1
B	0	1	1	1	1	0
A	1	0	1	0	0	1
B	1	1	1	2	1	0
B	1	0	1	0	1	1
B	0	1	1	1	1	0
B	0	1	1	1	0	1
B	1	0	1	0	1	0
B	1	1	1	2	1	0
A	0	0	1	3	0	1
B	0	0	1	3	1	0
B	1	0	0	3	1	0

NOT :	0	YOK
	1	VAR

NOT :	0*	Aktivasyon beklenmiyor.
	1*	Aktivasyon bekleniyor.
	2	Hem aktivatör hem inhibitör var
	3	Ligand-reseptörden biri eksik(bağlantı

*	Ligandı HLA-G olup bu kit ile tanımlanamıyor.
**	Ligandı HLA-A*03, HLA-A*11 olup bu kit ile tanıml

A-Bw4	HLA-A Kaynaklı (A23, A24, A25, A32,…) Bw4 ifade
	HER İNSANDA BULUNMASI GEREKENLER

2DS2	C1 GRUP	C1 aktivasyor	3DL1	3DS1	Bw4	A-Bw4	w4 aktivasyo
1	1	2	1	0	3	0	0
1	1	2	1	0	10	0	0
1	1	2	1	0	4	1	0
0	0	3	1	0	4	1	0
1	1	2	1	0	10	0	0
1	1	2	1	0	4	1	0
0	1	0	1	0	4	0	0
1	1	2	1	0	7	0	0
1	1	2	1	1	4	0	2
0	1	0	1	1	4	0	2
1	1	2	1	1	4	1	2
1	1	2	1	0	13	1	0
1	1	2	1	1	10	1	2
1	1	2	1	0	4	1	0
0	1	0	1	0	7	1	0
1	0	3	1	0	10	1	0
1	1	2	1	1	0	1	2
1	1	2	1	1	13	1	2
1	1	2	1	1	10	1	2
0	1	0	1	0	4	0	0
1	1	2	1	0	9	1	0
1	1	2	1	0	4	1	0
1	1	2	1	1	7	1	2
0	1	0	1	0	9	1	0
1	1	2	0	0	4	0	3
1	1	2	1	1	4	0	2
0	1	0	1	0	4	1	0
0	1	0	1	1	4	1	2
0	0	3	1	0	7	0	0
1	1	2	1	0	0	1	0
0	1	0	1	0	6	0	0
1	1	2	1	1	3	1	2
1	1	2	1	0	4	1	0
1	1	2	0	0	4	0	3
1	1	2	1	0	3	0	0

1	1	2	1	1	13	1	2
1	1	2	1	0	7	0	0
1	1	2	1	0	13	1	0
1	1	2	1	1	3	0	2
1	1	2	1	1	0	1	2
1	1	2	1	0	3	0	0
1	1	2	1	0	4	0	0
1	1	2	1	1	4	1	2
1	1	2	1	0	6	0	0
0	1	0	1	1	3	0	2
1	1	2	1	0	3	0	0
0	1	0	1	0	4	1	0
1	1	2	1	1	4	1	2
0	1	0	1	0	9	0	0
1	1	2	1	1	4	1	2
1	0	3	1	0	10	1	0
1	1	2	1	1	7	0	2
0	1	0	1	1	4	1	2
1	1	2	1	0	4	0	0
1	1	2	1	1	4	1	2
0	0	3	1	0	4	1	0
1	1	2	1	0	7	1	0
0	1	0	1	0	3	1	0

yok)

anamıyor.

eder.

2DL4	3DL2
*	**
*	**
*	**
*	**
*	**
*	**
*	**
*	**
*	**
*	**
*	**
*	**
*	**
*	**
*	**
*	**
*	**
*	**
*	**
*	**
*	**
*	**
*	**
*	**
*	**
*	**
*	**
*	**
*	**
*	**
*	**
*	**
*	**
*	**
*	**

	2DL1, C2grup	2DL2, C1grup	2DL3, C1grup	3DL1, Bw4
1	1	0	3	0
1	1	1	10	0
1	1	0	4	1
1	0	0	4	1
1	1	0	10	0
1	1	1	4	1
0	0	1	4	0
0	1	0	7	0
0	1	1	4	0
0	0	1	4	0
1	1	0	4	1
1	1	0	13	1
1	1	0	10	1
1	1	1	4	1
1	1	1	7	1
1	0	0	10	1
1	1	1	0	1
1	1	0	13	1
1	1	0	10	1
1	0	1	4	0
1	1	0	9	1
1	1	1	4	1
1	1	0	7	1
1	1	1	9	1
1	1	1	0	0
1	1	1	4	0
1	1	0	4	1
1	1	0	4	1
1	0	0	7	0
1	1	0	0	1
1	1	0	6	0
1	1	1	3	1
1	1	1	4	1
0	1	0	0	0
1	1	0	3	0

*	**
*	**
*	**
*	**
*	**
*	**
*	**
*	**
*	**
*	**
*	**
*	**
*	**
*	**
*	**
*	**
*	**
*	**
*	**
*	**
*	**
*	**
*	**

1	1	0	13	1
1	1	0	7	0
1	1	0	13	1
1	1	0	3	0
1	1	0	0	1
1	1	1	3	0
0	1	0	4	0
1	1	0	4	1
0	1	0	6	0
0	1	0	3	0
0	1	0	3	0
1	0	1	4	1
0	1	0	4	1
1	0	1	9	0
1	1	0	4	1
1	0	0	10	1
0	1	0	7	0
0	0	1	4	1
1	1	0	4	0
1	1	0	4	1
0	0	0	4	1
0	1	0	7	1
0	1	0	3	1

**AÇIKLAMA**	**0**	**YOK**
	**1**	**VAR**
	**3**	**Bw4 Thr80**
	**4**	**Bw4 Ile80**
	**6**	**Bw4 (Asp77**
	**7**	**Bw4 Thr80**
	**9**	**Bw4 Thr80**
	**10**	**Bw4 Ile80 +**
	**13**	**(Asp77+Thr**

NOT : **Ile80 hem İnhibitörler hem de A**

**3DL1, A-Bw4**	2DS1, C2grup	2DS2, C1grup	3DS1, Bw4
1	1	0	0
0	1	0	0
1	1	0	0
0	0	0	0
0	1	0	0
1	1	0	0
0	0	0	0
0	1	0	0
0	1	4	0
0	0	4	0
1	1	4	1
0	1	0	0
1	1	10	1
1	1	0	0
0	0	0	0
1	0	0	0
1	1	0	1
1	1	13	1
1	1	10	1
0	0	0	0
0	1	0	0
0	1	0	0
1	1	7	1
1	0	0	0
0	1	0	0
0	1	4	0
1	0	0	0
1	0	4	1
0	0	0	0
0	1	0	0
0	0	0	0
0	1	3	1
1	1	0	0
1	1	0	0
0	1	0	0

1	1	13	1
0	1	0	0
0	1	0	0
1	1	3	0
1	1	0	1
0	1	0	0
0	1	0	0
0	1	4	1
0	1	0	0
0	0	3	0
0	1	0	0
0	0	0	0
1	1	4	1
0	0	0	0
1	1	4	1
0	0	0	0
1	1	7	0
1	0	4	1
0	1	0	0
1	1	4	1
0	0	0	0
0	1	0	0
0	0	0	0

**7+Thr80)**
**+ Ile80**
**+ (Asp77+Thr80)**
**- (Asp77+Thr80)**
**80)**

ktivatörler için daha iyi ligand
